# Supplementary material for: Implementer and recipient perspectives of community-wide mass drug administration for soil-transmitted helminths in Kwale County, Kenya
Source: PLoS Negl Trop Dis. 2020 Apr 20;14(4):e0008258. doi: 10.1371/journal.pntd.0008258 (PMC7192516; doi:10.1371/journal.pntd.0008258)
Supplement: S1 List — (DOCX) [file pntd.0008258.s001.docx]

**List of abbreviations**

| CHA | Community Health Assistant |
| --- | --- |
| CHV | Community Health Volunteer |
| CIFF | Children’s Investment Fund Foundation |
| ComDT | Community directed treatment |
| EDCTP | European & Developing Countries Clinical Trials Partnership |
| FGD | Focus group discussion |
| IDI | In-depth interview |
| KEMRI | Kenya Medical Research Institute |
| LF | Lymphatic filariasis |
| LSHTM | London School of Hygiene & Tropical Medicine |
| MDA | Mass drug administration |
| M&E | Monitoring and Evaluation |
| MoH | Ministry of Health |
| NTD | Neglected tropical disease |
| PC | Preventative chemotherapy |
| PSAC | Preschool-age children |
| SAC | School-aged children |
| STH | Soil-transmitted helminthiases |
