# Supplementary material for: Implementer and recipient perspectives of community-wide mass drug administration for soil-transmitted helminths in Kwale County, Kenya
Source: PLoS Negl Trop Dis. 2020 Apr 20;14(4):e0008258. doi: 10.1371/journal.pntd.0008258 (PMC7192516; doi:10.1371/journal.pntd.0008258)
Supplement: Attachment — Submitted filename: PNTD-D-19-01749.docx [file pntd.0008258.s002.docx]

**Dear editor,**

In reference to the manuscript PNTD-D-19-01749 my opinion is rejected.

This work is of qualitative analysis, so the results obtained may be few useful for distant regions of Africa.

I believe that this kind of study should be presented in journals specialized in the subject with an anthropological and / or sociological approach.

Due to my specialty in quantitative analysis, I limit my analysis of the manuscript.

I emphasize that the work provides information on health policies related to STH but I do not consider it appropriate for this journal.

Best regards

Rubén Cimino
