# Supplementary material for: Implementer and recipient perspectives of community-wide mass drug administration for soil-transmitted helminths in Kwale County, Kenya
Source: PLoS Negl Trop Dis. 2020 Apr 20;14(4):e0008258. doi: 10.1371/journal.pntd.0008258 (PMC7192516; doi:10.1371/journal.pntd.0008258)
Supplement: Attachment — Submitted filename: Letter of Response 20200225 HL.docx [file pntd.0008258.s003.docx]

Response to reviewers for PLOS Neglected Tropical Diseases- PNTD-D-19-01749

We thank the reviewers for their thorough and thoughtful comments. Addressing these has undoubtedly strengthened the manuscript. We respond to specific comments below.

Methods

**Reviewer #2: Although the details are published elsewhere, I believe it would be relevant for readers to understand why the distribution occurred over an 8 day period, as this results in one of the major themes of the paper (increased workload by CHVs).**

Thank you, this is well noted. Typically, in Kenya the lymphatic filariasis programme delivers over a period of four days. Due to the fact that this was a research study and we were insisting on directly observed therapy and revisits to houses, it was decided in conjunction with the Ministry of Health at the National and County level to increase the time period, but not to create unsustainable expectations for future programmes. We have now included the text below to clarify how the 8-day period was decided on:

*“The distribution period of the MDA was decided upon in collaboration with the Ministry of Health and based on the timeline of previous MDAs in the study setting*”

**Reviewer #2: One of the themes that surfaced was that community members were more receptive to CHVs that were identified as leaders, however the methods did not include how CHVs were selected (were they selected from the same community? were they of the same religion? spoke the same language? male/female? previous employment (nurses or doctors?)) - may be good to mention in a sentence or two).**

We thank the reviewer for these valid observations and suggestions. We agree that more information could be provided on the terms of employment for the CHVs and have amended the manuscript with the following paragraph:

“*Delivery leveraged MoH community health service structures by recruiting existing frontline health workers, known as Community health volunteers (CHVs), as drug distributors. CHV selection was overseen by the County Public Health Office and organised by the Community Health Assistants (CHAs), according to standardised guidelines for selection, which included community participation in nomination of individuals. In all but a few exceptions CHVs were selected from the same community in which they lived and as a result were treating in their own village. They were required to cover approximately 120 households across eight days, and were paid using a performance-based strategy (500KSH per day – approximately USD$5.00 – consistent with nationally-recognized daily subsistence allowance rates). Terms of employment for CHVs were developed in consultation with the MoH and local government.*” 

**Reviewer #2: Throughout the study, what was the retention of CHVs and were the CHVs delivering the drugs to the same communities each time; and what was the impact of this on uptake and reach?**

The impact of retention on reach and uptake is an interesting issue. We know that less than 25% of CHVs took part in all four rounds of MDA in the TUMIKIA Project. However, unfortunately analysis of the impact of this is beyond the scope of this paper.

**Reviewer #2: The study conducted interviews 3 months after the third round of MDA -- is there potential recall bias because of the long period of time in between intervention and interviews?**

We thank the reviewer for this comment and recognise that there is the possibility of some recall bias due to the time gap between MDA and data collection. Although previous studies have used a similar time frame for data collection and we considered three months to not be an excessively long time period for respondents to recall perspectives and opinions concerning the activity [1], we have now included the possible issue of recall bias in the limitation section of the discussion:

*“It is possible that the three-month time period between MDA and data collection may have resulted in an increased occurrence of recall bias among respondents”*

Results

**Reviewer #1: there is very little that can be highlighted from this type of study.**

We respectfully disagree with the reviewer on this point and refer them to the discussion and conclusion sections which provide ample findings worth highlighting.  

**Reviewer #2: pg 13-- "Let them be trained and be recognized": in your summary of this major theme, consider emphasizing the CHVs sense of pride, obligation and duty towards their community -- perhaps include these words in your discussion. This sense of purpose is something program managers can capitalize on during recruitment.**

We note the observation and thank the reviewer for this suggestion but feel that sufficient weight is already given to highlighting this finding in both the results and discussion sections, see included text below:

*"*Implementers described factors that influenced their motivation to participate in the MDA and their intention to participate in future MDAs. Primary among these was their belief in the effectiveness of the intervention, which provided them with the opportunity to improve the health of their community, their sense of self-efficacy in carrying out their roles, and the level of financial and non-financial incentives, such as knowledge and recognition offered to them by participating in the intervention*”*

*“*Motivation to participate and continue to participate as a drug distributor was linked by respondents to belief in the effectiveness of the intervention*”*

**Reviewer #2: Line 271: I think that this quotation emphasizes a theme not mentioned directly, but very much so implied: continuous feedback from CHAs to program implementers on training and other issues-- how would this open and two-way communication flow work, and what would it look like? Consider adding this to your list of suggestions/ table, as you have illustrated, it is vital for MDAs.**

We agree with the reviewer that this is an interesting theme that has not been sufficiently referenced in the discussion section of the paper. We have added an item to our discussion table:

*“Develop adaptable implementation strategies that respond to feedback from/needs of implementers and recipients”*

And have made this addition to the main body of the discussion:

*“In addition to this, the findings point towards the need for active communication channels to be in place between program planners, implementers, and participants throughout the intervention that allow for stakeholder feedback to result in adaptations in MDA planning and delivery”.*

**Reviewer #2: Line 305: "drug distribution areas to be minimized" : could you clarify why they should be minimized? Wouldn't having more distribution areas (eg. one per community) make it easier for CHVs to deliver the drugs (instead of potentially having to transport them?)**

We thank the reviewer for this comment but think there may have been a misunderstanding here. The sentence is in reference to a suggestion of a minimisation of the travel time between storage points and distribution areas. One method of achieving this is, as you suggest, to increase the storage sites where the CHAs are based with the drugs so as to reduce the distance between the drug collection point and villages where they are distributing. However, as this was not specifically referred to by the participant, we did not include this as a specific suggestion.

**Reviewer #2: Discussion: Line 575: Absolutely love the clarity of the table, but found it rather redundant. Perhaps move the table into the results section, and eliminate some repetition from the discussion to make it more concise.**

We note the reviewer’s point here, but feel that the table would be unsuitable for the results section as it includes a column on recommendations, which are essentially synthesised discussion items, and not direct findings from the data. With regards to repetition, we agree that the table reproduces much of what can be found in the discussion and results, but due to the extremely wide scope of the study and the large number of themes present in the results, we felt the need for a table that succinctly tied the results, discussion and recommendations together across both the reach and uptake sections. 

**Reviewer #2: - I understand the concept of keeping themes broad for generalizability, but I think it would have relevant and interesting if specific myths or educational misnomers/themes were included and how this compared to similar studies (perhaps educational campaigns can target specific myths eg. safety during pregnancy, how they work etc).**

We agree with the reviewer that this is an interesting line of enquiry. As demonstrated in the text extracts below, several myths are brought out in the participants responses such as drugs used for contraception, drug safety, concerns over misuse such as witchcraft and poisoning.

*“Those who did not some said they heard rumors that the drugs are meant for family planning and they can cause strokes and elephantiasis”*

*“later they throw it, because the drug may be illuminati wanting to take their blood”*

*“They may be distributed by my enemy and he/she may poison me"*

We have added a sentence in the discussion that highlights that themes brought up here with regards to rumours and myths such as concerns of drugs used for contraception and fear of poisoning are common in MDA programmes:

*“The negative impact that rumours and fears surrounding side-effects can have on uptake has been extensively reported in the literature and this study adds further evidence that many of these myths, such as fears of covert contraception or poisoning, are common across different program and country settings”*

Conclusions


**Reviewer #3: The conclusions are somewhat depressing, as it appears almost impossible to effectively cover, find and treat whole communities. This is implicit in the conclusions.**

We thank the reviewer for this observation. As a point of reply we feel that the research offers concrete steps that PC-NTD programmes can take to improve reach and uptake during community-wide MDAs, and as such intend for it to be used to improve community coverage, rather than as evidence that high reach and uptake cannot be achieved.

**Reviewer #3: To this reviewer, the one omission is in the acknowledgements which omits the Kenya Government. If mass drug administration is to be applies (the paper talks of elimination), a major component will involve the Government, as there is clearly a need for national commitment and participation.**

Thank you for highlighting this omission. We agree that the Government indeed played a critical role and led the implementation of the MDA and will be the drivers of such initiatives moving forward. We have expanded on the section of the acknowledgements in which we thank the Kenyan Government, including the MOH and Ministry of the Interior and National Coordination and the Public Service Office:

“We would like to thank a number of key people for their valued input: Stefan Witek-McManus for input into development of the codebook and analysis plan; Janet Masaku and Rosemary Musuva for data coordination; and all the field officers, especially Hemed Mwakuzimui, Kennedy Kasbai, Naima Kamole and Dickson Muluu who conducted the transcription of the audio recordings. We appreciate the administrative and logistic support from Carlos Mcharo. We are very grateful to the Ministry of Health at the National level, particularly Sultani H Matendechero, Kefa Bota, Wycliffe Omondi, and Cecelia Wandera for oversight of the implementation of the mass drug administration (MDA). We also gratefully acknowledge Athuman Chiguzo, Hajara El-Busaidy, and Redempta Muendoat from the Ministry of Health in Kwale for their leadership during the implementation as well as the tireless work from the health teams at the County, Subcounty, health facility and village level teams. Furthermore, the MDA would not have been possible without the support of the Ministry of Interior and National Coordination of Government and the Public Service Office at the County, Subcounty, ward and village level. Finally, we thank the participants - CHAs, CHVs, and community members who took the time to provide valuable insights for this study.”

--------------------

Editorial and Data Presentation Modifications?

**Reviewer #2: Consider changing the word community "sensitization" to community education or engagement, as the sensitization process essentially involves a widespread educational campaign, and this wording has the potential to sound slightly condescending in global health settings.**

We agree that there are many potential words that could be used for this process, however in this case we would prefer to retain the term as we feel that the term sensitization is a widely used and understood term in the NTD community when referring to MDA programmes. 

**Reviewer #3: My main criticism is that the authors use acronyms and abbreviation. If they insist on this there should be a glossary at the beginning**

We agree that the manuscript contains a number of abbreviations and would be happy to provide a list of abbreviations if there is a specified location where this section could be included according to the editors.

Summary and General Comments

**Reviewer #2: I believe this article is extremely important for program implementers, logicians and others working in the field of global health. The information sheet for your study was provided in Swahili however interviews were conducted in many different languages. Further, was informed consent provided verbally due to potential illiteracy rates? Consider clarifying this information.**

We thank the reviewer for identifying this gap in information concerning informed consent. We have added the following line to the methods section to address this:

“*Illiterate participants or participants who did not speak Swahili had the consent sheet read to them in their preferred language by or in the presence of an independent, literate witness. The participant then either signed or provided a thumb print, observed by the witness who also provided their signature.* ”

References

1.	Peto TJ, Tripura R, Sanann N, Adhikari B, Callery J, Droogleever M, Heng C, Cheah PY, Davoeung C, Nguon C, et al: **The feasibility and acceptability of mass drug administration for malaria in Cambodia: a mixed-methods study.** *Trans R Soc Trop Med Hyg* 2018, **112:**264-271.
