# Supplementary material for: Implementer and recipient perspectives of community-wide mass drug administration for soil-transmitted helminths in Kwale County, Kenya
Source: PLoS Negl Trop Dis. 2020 Apr 20;14(4):e0008258. doi: 10.1371/journal.pntd.0008258 (PMC7192516; doi:10.1371/journal.pntd.0008258)
Supplement: Attachment — Submitted filename: Letter of Response 20200323.docx [file pntd.0008258.s004.docx]

Response to reviewers for PLOS Neglected Tropical Diseases- PNTD-D-19-01749

We thank the editor for their valued opinion and have made amendments to the manuscript based on their feedback. We respond to specific comments below.

**Item 1**

**REVIEWER #2: Consider changing the word community "sensitization" to community education or engagement, as the sensitization process essentially involves a widespread educational campaign, and this wording has the potential to sound slightly condescending in global health settings.**

**AUTHORS RESPONSE: We agree that there are many potential words that could be used for this process, however in this case we would prefer to retain the term as we feel that the term sensitization is a widely used and understood term in the NTD community when referring to MDA programmes.**

**EDITORS' OPINION:**

**I agree with Reviewer 2 that the term sensitization is not the best choice for the activities that took place, which were basically information dissemination strategies to try and engage the community. Although the term has been used a few times in the NTD/STH literature, it is NOT widely used and understood. Actually, there is no agreed upon definition. A paper by Dierick et al (2018) defines it as “…community sensitizations, which is a process whereby research staff arrange meetings to make information on the research available in the villages from which potential research participants may be recruited”**

**[Dierick et al. Community sensitization and decision‐making for trial participation: A mixed‐methods study from The Gambia. Developing World Bioeth. 2018;18:406–419. 10.1111/dewb.12160]**

**Even though the authors here elegantly refer to the engagement activities as “context-adapted sensitization strategy that leverages existing community structures and takes into consideration past community experiences of MDAs.” , in my opinion, community engagement would resonate better with the global health community.**

**On the other hand, sensitization was not seen by implementers as very well executed (“Implementers, for the most part, did not perceive sensitization activities to be effective in promoting community availability”). Similarly, sensitization was not enough to decrease mistrust among receivers: “While sensitization activities might adequately address perceived safety, efficacy and lack of knowledge, deep mistrust seemed to be a systemic problem”**

**I understand the reticence to accept this recommendation because the term sensitization is used more than 60 times in the manuscript. However, authors may want to keep in mind that “sensitization” is only the fourth “key actionable theme” emerging from data analysis, not the angular stone to MDA programs.**

We recognise the shared viewpoint of reviewer 2 and the editor on this issue and have adapted the manuscript to refer to ‘community engagement’ instead of ‘community-wide sensitization’, except in instances where we are referring to ‘“doorstep” sensitization’, which has been changed to ‘“doorstep” education’.

**Item 2**

**REVIEWER #2: Line 305: "drug distribution areas to be minimized" : could you clarify why they should be minimized? Wouldn't having more distribution areas (eg. one per community) make it easier for CHVs to deliver the drugs (instead of potentially having to transport them?)**

**AUTHORS RESPONSE: We thank the reviewer for this comment but think there may have been a misunderstanding here. The sentence is in reference to a suggestion of a minimisation of the travel time between storage points and distribution areas. One method of achieving this is, as you suggest, to increase the storage sites where the CHAs are based with the drugs so as to reduce the distance between the drug collection point and villages where they are distributing. However, as this was not specifically referred to by the participant, we did not include this as a specific suggestion.**

**EDITORS' OPINION:**

**The authors wrote: “CHVs emphasized the need for travel time between storage points and drug distribution areas be minimized”.**

**I can see why this wording can be confusing. To avoid it, I suggest rewording the sentence more simply to indicate that CHVs emphasized the need for reducing travel distance between storage points, which would result in maximizing resources and time utilization.**

This is well noted; we have amended the sentence in the manuscript to read as following:

“CHVs emphasized the need for travel distance between storage points to be minimized, highlighting that long distances from storage points to target communities resulted in increased time spent travelling or greater expenditure on transport.”

**Item 3**

**Reviewer #3: My main criticism is that the authors use acronyms and abbreviation. If they insist on this there should be a glossary at the beginning**

**AUTHORS RESPONSE We agree that the manuscript contains a number of abbreviations and would be happy to provide a**

**list of abbreviations if there is a specified location where this section could be included according to the editors**

**EDITORS' OPINION: it would be appropriate to provide a list of abbreviations at the end of the manuscript.'**

We thank the editor for this and have added a list of abbreviations to the end of the manuscript.
